# Validation of multiple single nucleotide variation calls by additional exome analysis with a semiconductor sequencer to supplement data of whole-genome sequencing of a human population

**DOI:** 10.1186/1471-2164-15-673

**Published:** 2014-08-10

**Authors:** Ikuko N Motoike, Mitsuyo Matsumoto, Inaho Danjoh, Fumiki Katsuoka, Kaname Kojima, Naoki Nariai, Yukuto Sato, Yumi Yamaguchi-Kabata, Shin Ito, Hisaaki Kudo, Ichiko Nishijima, Satoshi Nishikawa, Xiaoqing Pan, Rumiko Saito, Sakae Saito, Tomo Saito, Matsuyuki Shirota, Kaoru Tsuda, Junji Yokozawa, Kazuhiko Igarashi, Naoko Minegishi, Osamu Tanabe, Nobuo Fuse, Masao Nagasaki, Kengo Kinoshita, Jun Yasuda, Masayuki Yamamoto

**Affiliations:** Department of Integrative Genomics, Tohoku Medical Megabank Organization, Tohoku University, 2-1 Seiryo-machi, Aoba-ku, Sendai, 980-8573 Japan; Department of Biobank, Tohoku Medical Megabank Organization, Tohoku University, 2-1 Seiryo-machi, Aoba-ku, Sendai, 980-8573 Japan; Department of Applied Information Sciences, Graduate School of Information Sciences, Tohoku University, 6-6-05 Aramaki Aza Aoba, Aoba-ku, Sendai, Miyagi, 980-8579 Japan; Department of Biochemistry, Tohoku University Graduate School of Medicine, Seiryo-machi 2-1, Aoba-ku, Sendai, 980-8575 Japan; Department of Medical Biochemistry, Tohoku University Graduate School of Medicine, Seiryo-machi 2-1, Aoba-ku, Sendai, 980-8575 Japan; United Centers for Advanced Research and Translational Medicine, Tohoku University Graduate School of Medicine, 1 Seiryo-machi, Aoba-ku, Sendai, 980-8575 Japan; Institute of Development, Aging, and Cancer, Tohoku University, 4-1 Seiryo-machi, Aoba-ku Sendai, Sendai, 980-8575 Japan

**Keywords:** Next-generation sequencer, Population genetics, Whole-genome sequencing, Single nucleotide variations, Semiconductor-type sequencer

## Abstract

**Background:**

Validation of single nucleotide variations in whole-genome sequencing is critical for studying disease-related variations in large populations. A combination of different types of next-generation sequencers for analyzing individual genomes may be an efficient means of validating multiple single nucleotide variations calls simultaneously.

**Results:**

Here, we analyzed 12 independent Japanese genomes using two next-generation sequencing platforms: the Illumina HiSeq 2500 platform for whole-genome sequencing (average depth 32.4×), and the Ion Proton semiconductor sequencer for whole exome sequencing (average depth 109×). Single nucleotide polymorphism (SNP) calls based on the Illumina Human Omni 2.5-8 SNP chip data were used as the reference. We compared the variant calls for the 12 samples, and found that the concordance between the two next-generation sequencing platforms varied between 83% and 97%.

**Conclusions:**

Our results show the versatility and usefulness of the combination of exome sequencing with whole-genome sequencing in studies of human population genetics and demonstrate that combining data from multiple sequencing platforms is an efficient approach to validate and supplement SNP calls.

**Electronic supplementary material:**

The online version of this article (doi:10.1186/1471-2164-15-673) contains supplementary material, which is available to authorized users.

## Background

Whole-genome sequencing (WGS) of human genomic DNA with next-generation sequencers (NGSs) has opened a new avenue for personalized healthcare and personalized medicine based on the detection of genetic variations related to physical traits [1, 2]. The application of human WGS to large-population genetics requires rapid, low cost, and accurate validation technologies.

The resequencing market is currently dominated by Illumina HiSeq sequencing platforms (hereafter referred to as HiSeq) that have been applied in large population studies [3–5]. Bridging PCR amplification of fragmented genomic DNA in a flow cell and sequencing-by-synthesis chemical reactions truly realize massive parallel sequencing from both ends of a DNA fragment [6]. The output from a HiSeq instrument can reach up to 600 GB per run, with more than 80% of the reads with an average quality score higher than 30 (99.9% accurate). In particular, the newly released protocol for HiSeq (PCR-free library construction with rapid-run mode: 162 bp paired ends) omits the initial PCR amplification step during library construction and completes human WGS with high depth (up to 33×: 100 GBs) in two days in one flow cell. This improved protocol is expected to accelerate the discovery of disease-susceptible variants by the WGS analysis of human populations on a large scale.

Importantly, the accuracy of variant calls made with NGS data is critical for future genetic investigations that aim to detect disease-susceptible single nucleotide variations (SNVs) [7]. Even with the improvements in the chemistry used and in the equipment, systemic biases have been reported for both the genome coverage and the accuracy of variant calls of most NGSs [8]. Currently, the validation of SNV calls that are newly discovered using NGSs depends on conventional methods based on amplification of the target region with PCR, Sanger sequencing, hybridization of sequence-specific oligonucleotide probes, and mass spectroscopic assays [4, 8]. More than three million SNVs have been reported in a human genome compared with the reference GRCh37/hg19 sequence (http://www.ncbi.nlm.nih.gov/projects/genome/assembly/grc/) [4]. In the analyses of large populations, comprehensive validation of newly observed SNVs is sometimes prohibitively expensive and tedious using these traditional low-throughput methods.

It is of interest to discover whether the overall accuracy of variant calls can be improved using a hybrid approach such as using NGSs with different working principles to analyze the same genome, as indicated previously [4, 8]. The rationale of this notion is that platform-specific biases or errors in the data from one NGS platform can be corrected by using the data from another NGS platform, if the two methods are based on different working principles. We surmised, therefore, that an appropriate combination of different NGSs may reduce the overall cost of sequencing.

A semiconductor-based non-optical NGS has recently become available [9]. These sequencers are attractive candidates as alternatives to HiSeq. The semiconductor sequencers directly detect changes of pH that are caused by the release of hydrogen ions when nucleotides are incorporated into the growing DNA strand during the DNA polymerase reaction in cells within a chip, which is manufactured using the same technology that is used to construct integrated circuits [9]. This method features rapid reaction time and low price in consumables per base [10, 11]. The first semiconductor-based NGS, Ion Torrent Personal Genome Machine, has been used widely in many different applications [11–16]. The larger Ion Proton semiconductor NGS (hereafter referred to as Ion Proton) has now been launched, and the total output of the Ion Proton I chip is reported to be nearly 10 GB, which is suitable for targeted resequencing of, for example, the human exome. Because the sequencing reaction in semiconductor-based NGSs does not use terminator nucleotides, the accuracy of the generated reads are known to decrease for homopolymer repeat sequences [10, 12, 17–20]. Nonetheless, many known disease-causing mutations have been detected by the semiconductor sequencers [11, 12, 14, 15, 21], implying the potential of the platform.

It has been reported that a PCR-free protocol for HiSeq is not free from coverage bias, especially for high and low GC regions [17]. Therefore, the addition of exome data (generated using low-cost NGSs) to the WGS data processed by HiSeq may strike a balance between cost and benefit, making it an attractive strategy for sequencing functionally important regions in human populations. Here, we compare the variant call results for the genomic DNA of 12 Japanese individuals generated by WGS on HiSeq 2500 in rapid-run mode and targeted exome sequences obtained using on Proton with the I chip. We used the Omni 2.5-8 SNP arrays as references for the variant calls and compared the SNP data among the three platforms. We found that Ion Proton exhibited high concordance in variant calls with the other two platforms, indicating that Ion Proton is a promising tool for validation of multiple SNPs in the WGS of a large population.

## Results

### Outline of sequence outputs

A new sequencing protocol for HiSeq 2500, namely, a rapid-run mode with 162 bp paired-end sequences, was used in this study. A summary of the WGS data for the samples from 12 Japanese individuals produced by HiSeq 2500 is shown in Table 1. The average total number of bases and mapping ratio for the 12 samples were 101 GB and 95.7%, respectively (Table 1). The percent coefficient of variation (%CV) of total bases and mapping ratios among the 12 samples was small (Table 1), indicating that the quality of the sequence reaction was comparable among the samples. The automatic library construction using the Agilent Bravo liquid-handling robot contributed to the reproducibility among the samples. The %CV of quality scores (Q30) of the 100 to 150 cycles of READ 2 was a bit higher (10%), implying that the sequencing-by-synthesis technology may be less stable during the final one sixth of the cycles.

**Table 1:** **Basic statistics of the whole genome sequences generated by the HiSeq sequencer**

Samples	Total bases (GB)	Average depth	Read 2 %Q30: 100–150 cycles	Aligned bases (GB)	Mapping ratio (%)	SNPs
01	103	33.6	0.740	101	98	3,631,549
02	100	32.4	0.670	97.3	97	3,606,901
03	106	34.3	0.600	97.1	92	3,597,816
04	104	34.0	0.710	101	96	3,625,724
05	96	30.6	0.670	91.4	95	3,601,895
06	99	30.8	0.660	96.0	97	3,604,534
07	96	31.4	0.690	93.9	98	3,588,904
08	97	31.1	0.590	90.2	93	3,598,436
09	104	33.2	0.610	96.8	93	3,603,430
10	96	31.5	0.740	92.6	97	3,601,931
11	106	34.4	0.740	104	98	3,616,799
12	100	31.3	0.520	94.1	94	3,569,104
Mean	101	32.4	0.662	96.3	95.7	3603919
%CV	3.7	4.3	10	4.2	2.2	0.43

A summary of the whole exome sequencing data for the same 12 Japanese samples produced by Ion Proton is shown in Table 2. Two samples (01 and 03) did not meet our criteria for total number of bases (9 GB) with one Ion Proton I chip experiment; therefore, we repeated the Ion Proton run with samples from the same library and merged the two results before the variant calling (Table 2 and Methods). Although we used a 200 base read protocol, the average read length that we obtained was 140 (Table 2) because Ion Proton automatically trims unreliable bases [18]. We found that the variance in average read length was small compared with the variance in the total number of bases (p = 1.0 × 10^-17^, Student’s *t*-test), indicating that the sequencing reaction after the chip loading in Ion Proton was reproducible. Consequently, the %CV of the aligned bases was quite small (0.87%). The mean coverage depth of target sequences was 109× (Table 2), while the coverage depth may be somewhat overestimated because PCR duplication was not removed for the mapping. These results demonstrate that the HiSeq sequencing and Ion Proton sequencing both attained the standard quality.

**Table 2:** **Basic statistics of whole exome sequences generated by the Ion Proton sequencer**

Samples	Total bases (GB)	Average depth	Average read length (bp)	Aligned bases (GB)	Aligned bases (%)	SNPs
01_1	6.77	65.9	121	6.40	96	58037
01_2	8.37	85.1	127	7.90	95	
02	10.8	122	142	10.4	98	69667
03_1	8.24	78.5	144	8.00	97	67493
03_2	8.93	93.0	139	8.60	97	
04	11.2	126	149	10.8	97	53811
05	12.2	147	148	11.9	98	46923
06	9.25	94.5	149	9.00	98	52516
07	12.1	135	149	11.8	98	53993
08	10.7	103	138	10.3	96	52243
09	10.9	121	137	10.5	97	50166
10	11.6	136	142	11.3	97	50781
11	10.4	85.5	134	10.0	97	67810
12	11.3	133	146	10.9	97	54335
Mean	10.2	109	140	9.84	97.0	56481.25
%CV	15	23	5.9	16	0.87	12.94

Two independent runs (indicated as _1 and 2) were performed for Samples 01 and 03.

### Comparison of SNP calls among three platforms

To assess the potential of the Ion Proton exome sequencing to validate the SNV calls for the WGS generated by HiSeq 2500 of the 12 Japanese population samples, we evaluated the SNP calls using the Illumina Omni 2.5-8 SNP chip (hereafter referred to as Omni 2.5-8) as a reference to characterize the differences between the two NGS platforms. The reported Japanese genomic sequence generated by HiSeq 2000 seemed to be substantially different from the GRCh37/hg19 reference genome [22]. We focused on the autosomal target sequences of the Ion Proton exome kit (TargetSeq Exome Enrichment Kit, total target regions: 50 MB) to compare the NGS results from the two platforms. We extracted the variant call results, and tested the variants at the loci covered by Omni 2.5-8. A total of 79,143 SNPs on the autosomes in the 12 Japanese genomes were tested (Methods).

The numbers of called autosomal SNPs and the degrees of concordance among the three platforms (Omni 2.5-8, HiSeq, and Ion Proton) were the positivity of the alternate alleles, regardless of its allelic state (homozygous or heterozygous), from the GRCh37/hg19 reference genome. Omni 2.5-8 called 17,002 alternate alleles per person, either homozygous or heterozygous, on the 12 Japanese sequences and the %CV of the total calls of individuals was 0.54% (Table 3). The average number of SNPs called by the HiSeq software (16,858 SNPs) was fairly similar to the number called from Omni 2.5-8 with a similar variance (and 0.49%CV) (Table 3). Ion Proton called 15,637 SNPs with a relatively large variance (4.7%CV) among the 12 samples (Table 3). Therefore, we concluded that Ion Proton made less stable SNP calls among the individuals than HiSeq.

**Table 3:** **Concordant SNP calls made from the HiSeq, Ion Proton, and Omni 2.5-8 SNP array data**

Samples	Total calls	Omni 2.5	HiSeq	Proton	All concordant	HO concordant	HP concordant	OP concordant	Proton support
01	17031	16902	16782	15678	15549	16733	15576	15571	0.929
02	17168	17007	16872	14033	13879	16820	13896	13907	0.825
03	17156	17009	16860	15025	14855	16806	14889	14898	0.884
04	16964	16847	16712	15668	15552	16663	15580	15572	0.933
05	17029	16930	16782	16326	16208	16732	16238	16247	0.969
06	17151	17033	16873	15863	15723	16832	15744	15765	0.934
07	17193	17058	16918	16060	15905	16862	15939	15947	0.943
08	17223	17099	16939	16095	15935	16892	15965	15988	0.943
09	17282	17173	17017	16220	16084	16965	16114	16133	0.948
10	17030	16922	16800	16180	16067	16747	16098	16094	0.959
11	17086	16944	16802	14332	14182	16768	14199	14207	0.846
12	17234	17100	16938	16163	15996	16889	16028	16046	0.947
Average	17129	17002	16858	15637	15495	16809	15522	15531	0.922
%CV	0.56	0.54	0.49	4.7	4.8	0.49	4.8	4.8	4.7

HO concordant, HiSeq 2500 and Omni 2.5-8 chip SNP calls were concordant but not the Ion Proton calls. HP concordant, HiSeq 2500 and Ion Proton SNP calls were concordant but not the Omni 2.5-8 chip calls. OP concordant, Omni 2.5-8 chip and Ion Proton SNP calls were concordant but not the HiSeq 2500 calls. Calculation of proton support = All concordant/HO concordant.

Concordance between the Omni 2.5-8 and HiSeq calls was very high and much less variable among the samples (98.8–99.0% concordance) than the concordance between the Omni 2.5-8 and Ion Proton calls (81.8–96.0% concordance). The concordance among the calls by the three platforms exhibited relatively high variance compared with the concordance between the Omni 2.5-8 and HiSeq calls. The %CV is as low as 0.48% for the concordance between the calls made by Omni 2.5-8 and HiSeq. The variance in the numbers of concordant SNPs among the 12 individual genomes seems to be caused by fluctuations in the number of SNPs (14,033–16,220) called by Ion Proton (Table 3).

**Figure 1:**
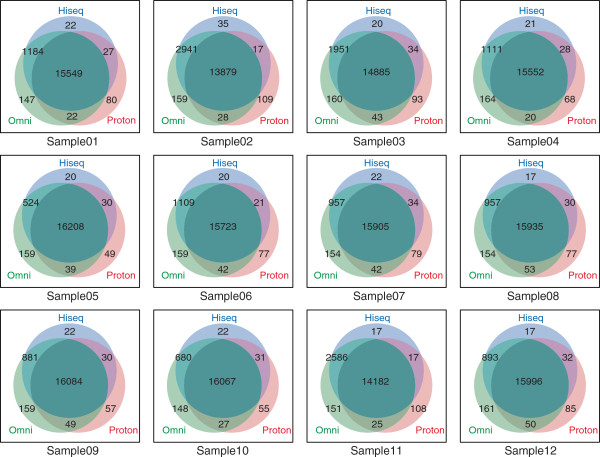
**Venn diagram of SNP calls for each sample shared among the three platforms.** The numbers in each area indicate the number of SNPs. The size of the area is not proportional.

Venn diagrams of the SNP calls shared among the three platforms are shown in Figure 1. A maximum of 97% of the HiSeq calls for any one individual were supported by the Ion Proton calls (see also Table 3). Indeed, the numbers of concordant SNPs including “negative” calls (SNP calls that corresponded to reference genome sequences) were nearly 98% (Figure 1 and Additional file 1: Table S1). The overall concordance of SNP calls made by HiSeq and Ion Proton with calls made by Omni 2.5-8 was 99.6% and 97.5%, respectively (Additional file 1: Table S1). These numbers are comparable with similar results reported in a previous study [10]. While almost 200 of the called SNPs were discordant between Omni 2.5-8 and both HiSeq and Ion Proton (Additional file 1: Table S1), many of these SNP calls seemed to be derived from a difference in the allelic calls in the manifest file of Omni 2.5-8 [10]. These results support the promising potential of combining the NGS platforms as a multiple validation method for SNV calls in population studies.

### Reproducibility of SNP calls by individual NGSs

**Figure 2:**
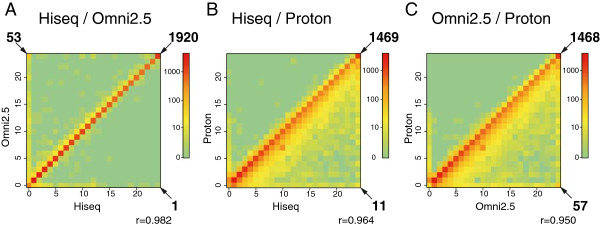
**Level plots of alternative allele counts among the three platforms.** Scatter plots of alternative allele counts between **A)** HiSeq and Omni 2.5-8, **B)** HiSeq and Proton, and **C)** Proton and Omni 2.5-8 are indicated. The vertical and horizontal axes show the numbers of alternative allele calls for each platform. The color bar indicates the number of SNPs in a pixel. There were 12 individual samples, so the maximum number of alternative alleles is 24. The digits with arrows near the corner indicate the numbers of SNPs at the corner pixels.

To analyze the reproducibility of SNP calls among the three platforms in detail, a level plot of the numbers of alternative allele calls in the 24 alleles (12 individual samples with two possible alleles for each SNP) was generated (Figure 2). If all the SNP calls are completely identical between two of the three platforms, the data points accumulate on the 1:1 diagonal line. For the HiSeq and Omni 2.5-8 SNP calls, the points accumulated along the diagonal line, indicating very high concordance between these two platforms (Figure 2A). For both the HiSeq and Omni 2.5-8 SNP calls against the Ion Proton calls, the plot showed that Ion Proton tended to call a lower number of SNPs than HiSeq and Omni 2.5-8 (Figure 2B and 2C). However, we also found that only a small fraction of alternate alleles in the 12 Japanese samples at one of the SNP loci was never called by Ion Proton (horizontal axes of Figure 2B and 2C).

The level plot analysis also indicated possible systemic errors inherent in each platform. For example, 53 SNPs were not called by HiSeq for any of the 12 samples, whereas all of them were called as homozygous for the alternate alleles by Omni 2.5-8 (Figure 2A, upper left corner). Visual inspection of the BAM files using the Integrated Genome Viewer [23, 24] suggested that some of the SNP loci may be caused by the large structural variations that are commonly found in the genomes of Japanese populations, implying that population-specific reference genomic sequences will improve the quality of mapping of NGS reads to reference genomes.

To detect systemic errors in the SNV calls, we analyzed the commonly discordant SNPs among the 12 individuals (Table 4). On average, Ion Proton called 1,849 SNPs that exhibited discordant variant calls in an individual exome, and 43 (2.3%) of them were commonly discordant among the 12 Japanese genomes. HiSeq called an average of 64 SNPs that were discordant with Ion Proton and Omni 2.5-8, suggesting that Ion Proton may cover some calls that were missed by HiSeq. Together, these results indicate that the two NGS platforms showed no strong systemic calling biases for the SNPs covered by Omni 2.5-8.

**Table 4:** **Average numbers of NGS SNP calls showing concordant or discordant with Omni 2.5-8**

	Proton concordant & HiSeq discordant	HiSeq concordant & Proton discordant	All concordant	Both discordant		
				Total	HiSeq = Proton	All discordant
Average	64	1849	76945	216	202	14
%CV	19.6	55.6	1.24	4.23	5.51	31.6
Common	1	43	66472	80	67	1

### Effects of sequence depth and GC contents on SNP calls by the NGSs

**Figure 3:**
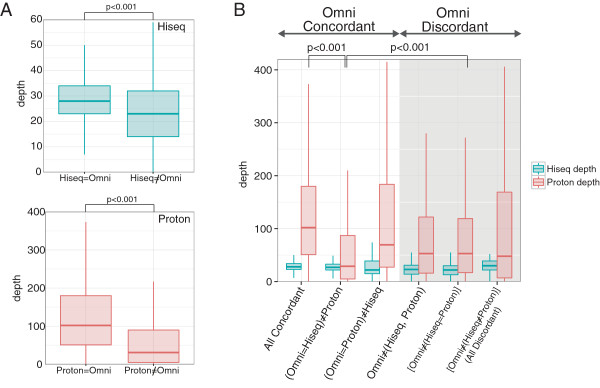
**Box plots of read depths at concordant/discordant SNP loci among the NGSs and Omni 2.5-8. A)** Box plots of the NGS read depth at the SNP loci showing concordance/discordance between the NGSs and Omni 2.5-8. The box indicates the first and third quartiles and the lines indicate the highest and lowest value that is within 1.5 x inter-quartile range. Outliers were omitted. The graph on the left indicates the distribution of read depths at HiSeq2500 SNP loci and the graph on the right indicates the distribution of read depths at Ion Proton and Omni 2.5-8 SNP loci. **B)** Box plots of the NGS read depths at the SNP loci showing concordance/discordance among the three platforms. The box indicates the first and third quartiles and the lines indicate the highest and lowest value that is within 1.5 x inter-quartile range. Outliers were omitted. The three columns (shaded gray rectangle) from right indicate the distribution of read depths of the discordant SNPs between the NGSs and Omni 2.5-8. The read depth at SNP loci that showed Ion Proton discordance against HiSeq 2500 and Omni 2.5-8 was significantly different. The read depths at SNP loci for SNPs that both HiSeq 2500 and Ion Proton failed to call are shown in the gray box.

**Figure 4:**
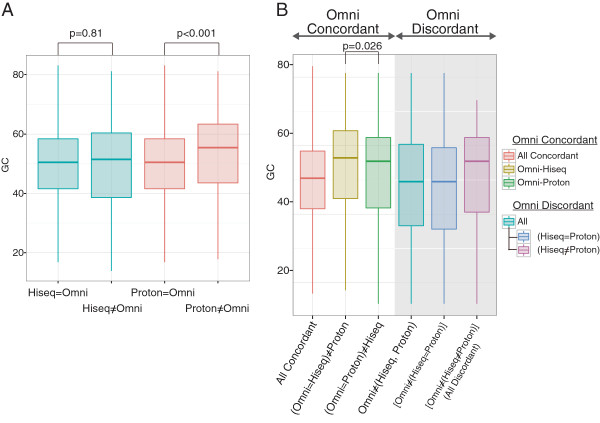
**Box plots of GC content at concordant/discordant SNP loci among the NGSs and Omni 2.5-8. A)** Box plots of the GC content at the SNP loci showing concordance/discordance between the NGSs and Omni 2.5-8. The box indicates the first and third quartiles and the lines indicate the highest and lowest value that is within 1.5 x inter-quartile range. The graph on the left indicates the distribution of GC content at HiSeq SNP loci. There was no statistically significance difference in GC content between the concordant and discordant SNPs (p = 0.81). The graph on the right indicates the distribution of GC content at Ion Proton and Omni 2.5-8 SNP loci. The difference in GC content between concordant and discordant SNPs was significant (p = 2.2 × 10^-16^). **B)** Box plots of the GC content at the SNP loci showing concordance/discordance among the three platforms. The three columns (shaded gray rectangle) from right indicate the distribution of GC content of the discordant SNPs between the NGSs and Omni 2.5-8. The GC content at SNP loci that showed Ion Proton discordance compared with HiSeq and Omni 2.5-8 was significantly different (p = 0.026).

Read depth, GC content, and homopolymer stretches have been identified as critical factors for the accuracy of SNV calls made by the NGSs [17, 20, 25]. For HiSeq, the read depth at each SNP was significantly different between the concordant and discordant calls with Omni 2.5-8 (p < 0.001, Mann–Whitney U test) as well as with Ion Proton (p < 0.001, Mann–Whitney U test, Figure 3A). Similarly, the variant calls discordant with Ion Proton exhibit significantly lower read depth (p < 0.001, Mann–Whitney U test, Figure 3B). These results confirm the previous observations that read depth can be a major factor in determining the quality of variant calls. As mentioned earlier, we did not eliminate duplicate reads from the Ion Proton data. The ratio of duplicated reads at SNVs in the Ion Proton reads for each of the 12 samples was found to be inversely correlated to the concordance of SNP calling comparing with the Omni 2.5-8 (Additional file 2: Figure S1).The GC content was calculated in a 101-base window for each SNP locus (50 bases on each side). We found that Ion Proton calls were affected in the loci with higher GC content (Figure 4). For the HiSeq SNP calls, no significant difference in the GC content of the loci was detected between the SNPs concordant and discordant with Omni 2.5-8, whereas, for the Ion Proton calls, some significant difference was found (p < 0.01, Figure 4A). Similarly, the concordant SNPs between Ion Proton and Omni 2.5-8 tended to have higher GC content than the discordant SNPs (Figure 4B). These results indicate that GC content in the SNP loci affected the quality of SNP calls by the NGSs, especially for Ion Proton.

**Figure 5:**
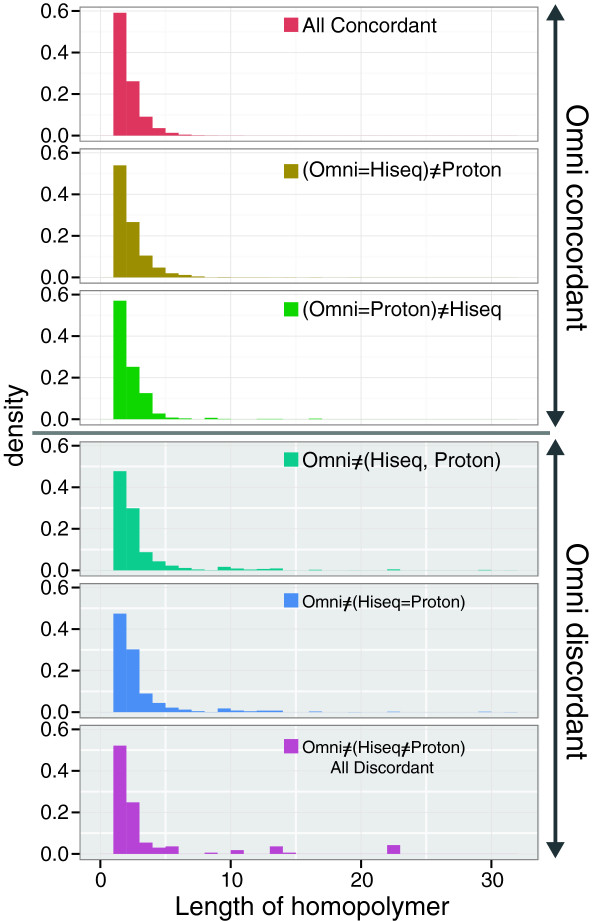
**Distribution of homopolymer lengths at concordant/discordant SNP loci among the three platforms.** The vertical axis of each panel indicates the density of each homopolymer at the SNP loci, calculating the total number of SNPs for each category as 1. The horizontal axis of each panel indicates the length of the homopolymers.

It has been noted previously that semiconductor sequencers frequently show insertion/deletion (indel) errors at loci with homopolymer sequences [17–19]. However, against our expectation, homopolymer length in the SNP loci did not affect the number of discordant SNPs between Ion Proton and Omni 2.5-8 (Figure 5), suggesting that SNPs in short homopolymer stretches can be detected by Ion Proton.

Logistic regression analyses indicated that read depth was the strongest factor for discordant calls against Omni 2.5-8 for both HiSeq and Ion Proton (Table 5); however, GC content affected more Ion Proton calls (2.5 fold) than HiSeq calls. In contrast to the effect of homopolymer sequences on discordant SNP calls, the logistic regression analysis results showed that homopolymer sequences in the SNP loci effected Ion Proton variant calls more strongly than they affected HiSeq variant calls, even though the overall effects were not significant (Table 5 and Figure 5). These results indicate that all three factors (read depth, GC content, and homopolymer stretches) may affect the accuracy of the Ion Proton SNP calls, whereas read depth may be the major factor for the accuracy of the HiSeq SNP calls.

**Table 5:** **Logistic regression analysis among QC factors of NGS and calling discordance with Omni 2.5-8**

Platform	A	B	C	A + B	A + C	A + B + C
	Read depth	GC contents	Homopolymer			
HiSeq 2500	0.0854	0.0021	0.000074	0.0896	0.104	0.106
Ion Proton	0.0759	0.0051	0.0123	0.0837	0.0759	0.0839

In addition, we calculated the transition/transversion ratio for Omni 2.5-8 and the two NGSs and found that the transition/transversion ratio was smaller for variant calls between the two NGSs but not between either of the two NGSs and Omni 2.5-8 or are all discordant SNPs, indicating that the NGSs might miss more transitions than transversions (Additional file 1: Table S2). The transition/transversion ratio values that we obtained are compatible to the ratios reported in a previous study [26].

## Discussion

In this paper, variant SNP calls in the exonic sequences from the genomic DNA of 12 Japanese individuals were compared between two NGS platforms, targeted exome sequencing by Ion Proton and PCR-free WGS by HiSeq 2500, and Omni 2.5-8. The Omni 2.5-8 chip SNP calls were used as the reference. Approximately 80,000 SNP loci were analyzed in this study from which Omni 2.5-8 called 17,000 variants, and more than 90% of these variant calls were supported by both HiSeq and Ion Proton. Ion Proton exhibited a maximum of 96% concordance of SNP calls with the SNP calls by the other two platforms, indicating that Ion Proton is a promising tool for the valiadation of multiple SNPs. Nonetheless, improvements in the sensitivity of Ion Proton for alternative alleles are still necessary for the efficient detection of novel SNVs in a human genome using Ion Proton.

Of the three factors that are known to affect SNV calling by NGSs, we found that all three factors (read depth, GC content, and homopolymer length) affect more or less the accuracy of Ion Proton SNP calls, whereas read depth was the major factor that affected HiSeq SNP calls. GC content has been shown to be a confounding factor for exon capturing [27–29]; therefore, the accuracy of Ion Proton SNP calls may have been affected by differences in the GC content near the SNP loci. Non-optical semiconductor-based sequencers like Ion Proton have been used widely for multiple amplicon sequencing [11–13, 30–35]; however, it has been reported that these sequencers exhibit relatively high error rates, especially for homopolymer sequences [17–19, 36]. While homopolymer length did affect the accuracy of the Ion Proton SNP calls more than it affected the accuracy of the HiSeq SNP calls, its affect on the Ion Proton SNP calls was small (the logistic regression coefficient for homopolymer length in Ion Proton was 0.0123, Table 5).

An interesting question is whether Ion Proton can cover errors found in HiSeq data. We found that HiSeq failed to call from 22 to 53 SNP calls in the 12 individual samples, which were called commonly by Ion Proton and Omni 2.5-8 (Figure 1), indicating that Ion Proton may cover the HiSeq missed calls. The Ion Personal Genome Machine (PGM) that preceded Ion Proton was used for a similar purpose in the characterization of the Tasmania devil genome sequence [37].

We found that there was a significant difference between the number of SNPs called among the 12 samples by HiSeq and Ion Proton, and the variance was larger for Ion Proton calls than it was for HiSeq calls. The sequencing processes in Ion Proton after the chip loading appeared to exhibit reproducibility similar to that of HiSeq during sequencing-by-synthesis. Therefore, the major reason for the variance in the number of Ion Proton SNP calls must be in the different library construction procedures that are used in the two NGSs. In this study, the library preparation procedures that we used for Ion Proton were manual, whereas liquid-handling robots were used for the construction of libraries for HiSeq. We surmise that the Ion Proton output will improve if automated liquid-handling procedures can be established. Indeed, we found that the reproducibility of library construction for Ion Proton was improved using an automated library construction method (SS, INM, and JYa: unpublished observation).We found that most of the SNPs called by HiSeq were supported by the Ion Proton SNP calls; indicating that Ion Proton could be used to validate novel SNPs in a population. However, if the SNPs are relatively rare in a population, they may not be validated by Ion Proton. In fact, the pixels near the origin on the horizontal axis are a bit brighter than those farther from the origin, as can be seen in Figure 2B and 2C. The data plotted in Figure 2B and 2C demonstrated how the addition of Ion Proton SNP call data to HiSeq SNP calls can provide strong support for the identification of alternate alleles in a population of interest. Our results suggest that approximately 97% of the expected SNVs in exonic regions of a human genome will be verified by Ion Proton exome sequencing. This finding will contribute to designing custom arrays for large-scale population-specific genetic studies of populations of unique origin, like the Japanese.

## Conclusions

Validation of SNVs detected in WGS is critical for studying disease-related variations in human population genetics. Combining different types of NGSs in analyses of the genome sequences from the same individual may be an efficient means of validateing multiple SNV calls simultaneously. Here, we attempted to show the versatility and usefulness of the combination of Ion Proton exome sequencing with HiSeq 2500 WGS by analyzing 12 independent Japanese genomes sequences and comparing the corresponding SNP calls, with the Omni 2.5-8 SNP calls as the reference. We found that Ion Proton exhibited a maximum of 97% concordance in variant calls with the other two platforms, indicating that Ion Proton is a promising tool for the validation of multiple SNPs in the exons of genomic sequences.

## Methods

### Specimens

The Tohoku Medical Megabank Organization (Tohoku University Graduate School of Medicine, Miyagi, Japan) constituted the prospective cohort (design paper is in preparation), and 5 ml of peripheral blood was donated by the Japanese participants, all with written informed consent. The whole project was approved by the ethical committee of the Tohoku University School of Medicine. Because of their availability for further follow-up investigations, we selected the first 12 participants of the cohort (10 males and 2 females) who lived in the neighbor of Tohoku Medical Megabank Organization.

### DNA preparation

To extract the genomic DNA, the whole peripheral blood samples were processed with an Autopure LS system (Qiagen, Germany) for automated nucleotide purification following the manufacturer’s instructions. We omitted the RNase treatment, measured the concentration of the double-stranded DNA with PicoGreen (Life Technologies, Carlsbad, CA), and adjusted the concentration of the DNA to 200 ng/μL in Elution Buffer (Qiagen, Germany).

### PCR-free whole-genome sequencing with the HiSeq 2500 sequencer

The genomic DNA (2 μg in 100 μL) was sonicated using a Covaris LE220 (Covaris Inc., Woburn, MA) to an average target size of 550 bp. The sheared DNA was used for library construction with the TruSeq DNA PCR-free sample preparation kit (Illumina, San Diego, CA) on a Bravo liquid-handling instrument (Agilent Technologies, Santa Clara, CA). The libraries were analyzed using a DNA Fragment Analyzer (Advanced Analytical Technologies, Ames, IA) and quantified by real-time PCR using the KAPA Library Quant Kit (KAPA Biosystems, MA).

Ten microliters of 2 nM libraries were denatured with an equal volume of 0.1 N sodium hydroxide; 1.5–2.0 pM of the denatured library was then used for on-board cluster generation on a HiSeq 2500 system (Illumina) with a TruSeq Rapid PE Cluster Kit (Illumina). Then the sequencing-by-synthesis reaction was performed in rapid-run mode with a 162-bp paired-end protocol. We ran one sample per flow cell so that no index read was needed. For each reaction, one and a half TruSeq Rapid SBS (sequencing-by-synthesis) kits (200 cycle) were used.

### Preparation of libraries for TargetSeq exome capture

Genomic DNA was prepared for exome capture according to the protocol included with the Ion Xpress Plus Fragment Library Kit for the AB Library Builder (Life Technologies, Carlsbad, CA) using the AB Library Builder System (Applied Biosystems, Foster City, CA). The DNA solvent was exchanged with pure water by ethanol precipitation. Enzymatic shearing was performed using 1–2 μg genomic DNA per sample. Sheared DNA was purified using the Agencourt AMPure XP Reagent (Agencourt, Boston, MA) with a target size peak of 350 bp, followed by adaptor ligation (A1 and P1). The adaptor-ligated genomic DNA fragments were then eluted in 45 μL of low TE buffer and amplified by PCR using 200 μL of Platinum PCR Supermix High Fidelity (Life Technologies), 5 μL of 50 μM library amplification primer mix, and 45 μL ligated DNA. The thermal cycling protocol was initial denaturation at 95°C for 5 min, followed by 7–8 cycles at 95°C for 15 s, 58°C for 15 s, and 72°C for 60 s. The amplified library was purified and eluted in 50 μL of low TE buffer using AMPure XP reagent.

### Exome capture

Pre-capture library DNA was hybridized to exome capture probes using an Ion TargetSeq Exome Enrichment Kit (Life Technologies) according to the manufacturer’s specifications. A total of 500 ng of library DNA, 5 μL of 1 mg/mL Human Cot-1 DNA, and 5 μL of Ion TargetSeq Blocker P1 and A per sample were mixed and dried using a CC-105 centrifugal concentrator (TOMY, Tokyo, Japan) at the high temperature setting for 40 min. The dried DNA was dissolved in 7.5 μL of TargetSeq Hybridization Solution A, added to 3 μL of Enhancer B and 4.5 μL of Exome Probe Pool, and hybridized at 47°C for 72 hours in a Veriti Thermal Cycler (Applied Biosystems, Foster City, CA). The probe-hybridized library DNA was enriched by binding to streptavidin-coated M-270 beads (Dynal, Oslo, Norway), rotated at 650 rpm at 47°C for 45 min in a Thermomixer comfort (Eppendorf, Hamburg, Germany). The streptavidin conjugate was washed with TargetSeq Hybridization and Wash Kit solutions (Life Technologies) strictly according to the manufacture’s protocol and eluted in 30 μL of DNase-free water. The probe-hybridized DNA was further amplified by PCR using 200 μL of Platinum PCR Supermix High Fidelity, 20 μL of library amplification primer mix, and 30 μL of the probe-hybridized DNA. The thermal cycling protocol was initial denaturation at 95°C for 5 min followed by 12 cycles at 95°C for 15 s, 58°C for 15 s, and 72°C for 60 s. The amplified exome DNA library was subjected to size selection using E-Gel SizeSelect Agarose Gel (Applied Biosystems), purified twice with a 1.5-fold volume of AMPure XP reagent, and eluted in 25 μL of low TE buffer. The quantity and quality of the captured libraries were assessed using a StepOne Plus qPCR instrument (Life Technologies) with an Ion Library Quantitation Kit (Life Technologies) and Bioanalyzer instrument (Agilent Technologies, Santa Clara, CA) with Agilent High Sensitivity DNA Kit (Agilent Technologies) according to the manufacturers’ instructions.

### Template preparation and sequencing with an Ion Proton sequencer

Emulsion PCR was performed using a OneTouch 2 instrument (Life Technologies) with an Ion PI Template OT2 200 Kit v2 following the manufacturer’s instructions. The enrichment of template-positive Ion Sphere Particles (ISP) in the Ion Proton I chip was achieved using the Ion OneTouch ES enrichment system (Life Technologies). Ion Proton I chip version 2 was prepared and loaded according to the manufacturer’s recommendations. The total base output as a criterion for a successful experiment was set as 9 GB. If a sample did not reach this criterion for the total base output in one experiment, we performed the sequencing again with the same library and merged the results before aligning the reads to the reference GRCh37/hg19 sequence.

### SNP array scanning with the iScan system

We used a Human Omni 2.5-8 v1.1 DNA Analysis Kit (Illumina) to analyze 160 ng of genomic DNA following the manufacturer’s instructions. In brief, the genomic DNA was subjected to isothermal amplification followed by fragmentation with nuclease. The DNA was precipitated with 2-propanol, then hybridized with oligonucleotide probes immobilized on Human Omni 2.5-8 BeadChips (eight samples per BeadChip slide). After washing, the probes underwent single-base extension using the captured genomic DNA as templates and incorporating 2, 4-dinitrophenyl- or biotin-labeled nucleotides to identify the genotypes. Then, immunohistochemical staining was performed to amplify the incorporated signal. Two Robotic Universal modules (Freedom evo, TECAN, Maennedorf, Switzerland) and the Illumina Infinium LIMS system (Illumina) were used in a series of experiments. An iScan scanner system with AutoLoader 2.X controlled by iScan Control Software ver. 3.3.28 (Illumina) was used for data acquisition. The SNP calling was performed using the Genotyping Module in the GenomeStudio software (ver.2011.1: Illumina). The default set cluster file was HumanOmni2-5 M-8b1-1_B.egt (Illumina), and a Gen Call Threshold of 0.15 was used for SNP calling. The SNP call rate was calculated and samples with overall call rates over 99% and LogRdev values below 0.2 were used for further analysis.

### Variant calling pipeline for Illumina HiSeq sequencing

Fastq files were generated by base calling with CASVA 1.8.2. Reads in the generated fastq files were mapped to the human reference genome (GRCh37/hg19) with decoy sequences (hs37d5) using the BWA-MEM alignment algorithm in BWA version 0.7.5a-r405 [38, 39] with the default options, and stored as BAM files. The following post-processing was applied to the BAM files: reads in the BAM files were realigned with Realigner Target Creator and Indel Realigner in the Genome Analysis Tookit 2.5-2 (GATK), and their base quality scores were recalibrated with Base Recalibrator and Print Reads in GATK [40, 41]. For the Realigner Target Creator and Indel Realigner, no VCF file of known indel sites was given as input. SNP sites in NCBI’s SNP database (dbSNP, version 137) in a VCF file were input to Base Recalibrator as known SNP sites. Variant calling was conducted with the post-processed BAM files using Unified Genotyper in GATK with the default options [40, 41], and the results were stored in VCF files.

### Variant calling pipeline for Ion Proton sequencing

The variant calling pipeline of Life Technologies was used to analyze the Ion Proton sequencing runs. Reads were aligned to the GRCh37/hg19 reference sequence using tmap software version 3.6.39. Variant discovery and genotype calling of multi-allelic substitutions and indels was performed on each individual sample using the Torrent Variant Caller (TVC) version 3.6.39. Parameters for variant discovery were set based on the “TVC 3.6 Parameters for TargetSeq Exome on Proton PI” with thresholds (snp_beta_bias = 150, snp_strand_bias = 0.9999, maximum common signal shift = 0.5, snp_min_variant_score = 5, and minimum_mapping _quality_score = 0) changed from the default values suggested by Life Technologies to use as many reads as possible.

### Analysis tools and selection of SNPs

To compare the genotypes from the three platforms, the output VCF files from the two NGS outputs and the Omni 2.5-8 output files formatted with PLINK [42] were processed to generate subsets that contained the common target SNP sites. The common target SNP sites was obtained by intersecting autosomal target manifests, Ion-TargetSeq-Exome-50 Mb-hg19.bed by Life Technologies, and the bed file of Omni 2.5-8 from the Human Omni 2.5-8 v1.1 DNA Analysis Kit by Illumina, using the BEDTools software suite [43].

SNPs outside of the common target SNP sites were filtered out leaving 83,237 sites as the common targets. From these loci, six probes for detection of copy number variations were removed. We also found 2,626 overlapping probes in the Omni 2.5-8 array and integrated the SNP calls using these corresponding probes. In total, we analyzed 79,143 SNPs.

Genotyping data on each platform were obtained from the VCF files and PLINK/PED files using a set of in-house scripts; then, the genotype concordance and accuracy were calculated.

### Statistical analyses

Differences in the NGS read depth between discordant and concordant SNP calls with the Omni 2.5-8 calls were examined using the Mann–Whitney U test with SAMtools [44], in-house scripts, and the R statistical environment [45]. Logistic regression analyses for the discordant and concordant calls between the NGSs and Omni 2.5-8 with three NGS quality control (QC) parameters (read depth, GC content, and homopolymer length) were performed in the R statistical environment. Logistic regression analysis of the SNPs with read depths in the range of ± 1 SD from the average was performed.

### Data availability

The SNP calls at each position covered by Omni 2.5-8 are attached as Additional file 3 (for HiSeq 2500), Additional file 4 (for Proton), and Additional file 5 (for Omni 2.5-8). Genomic DNAs used in this study will be distributed through Tohoku Medical Megabank Organization upon request.

## Electronic supplementary material

Additional file 1: **Concordant/discordant SNPs and transition/transversion ratios for Omni 2.5-8 and the two NGSs.** This file contains Tables S1 and S2 as Excel Spreadsheets. (XLS 36 KB)

Additional file 2: **Effect of duplicated reads in the Ion Proton SNP calls.** This file contains Figure S1 in PDF format. The duplication rate of Ion Proton reads was calculated for each sample using the BamDuplicates module of the Torrent Suite 4.0 (Life Technologies). The vertical axis indicates the mean duplication rate and the horizontal axis indicates the concordance rate of variant calls between Omni 2.5-8 and Ion Proton for each sample. (PDF 130 KB)

Additional file 3: **The VCF file of merged SNP calls by HiSeq2500.** (ZIP 323 KB)

Additional file 4: **The VCF file of merged SNP calls by Ion Proton.** (ZIP 291 KB)

Additional file 5: **The VCF file of merged SNP calls by Omni 2.5-8.** These files contain SNP information corresponds to the Omni 2.5-8 probe set found in the target exons of Ion TargetSeq Exome Enrichment Kit. (ZIP 217 KB)
