# Supplementary figures and images for: Validation of multiple single nucleotide variation calls by additional exome analysis with a semiconductor sequencer to supplement data of whole-genome sequencing of a human population
Source: BMC Genomics. 2014 Aug 10;15(1):673. doi: 10.1186/1471-2164-15-673 (PMC4138778; doi:10.1186/1471-2164-15-673)

Supplementary Figure 1

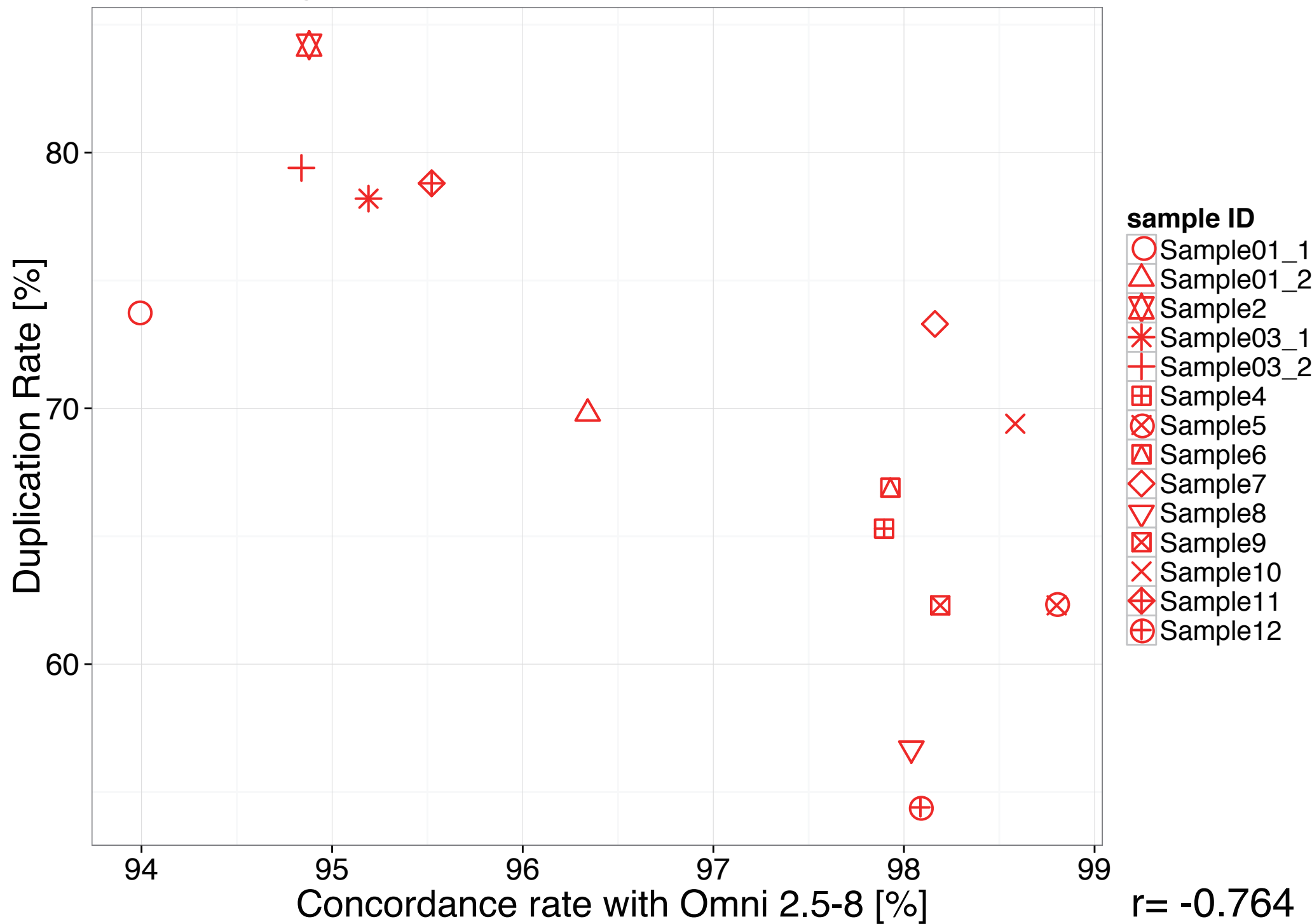

Supplement: Supplementary file 2 — Additional file 2: Effect of duplicated reads in the Ion Proton SNP calls. This file contains Figure S1 in PDF format. The duplication rate of Ion Proton reads was calculated for each sample using the BamDuplicates module of the Torrent Suite 4.0 (Life Technologies). The vertical axis indicates the mean duplication rate and the horizontal axis indicates the concordance rate of variant calls between Omni 2.5-8 and Ion Proton for each sample. (PDF 130 KB) [file 12864_2013_6358_MOESM2_ESM.pdf]
